# Nutrient and ruminal fermentation profiles of *Camellia* seed residues with fungal pretreatment

**DOI:** 10.5713/ajas.18.0612

**Published:** 2018-10-26

**Authors:** Chunlei Yang, Zhongfa Chen, Yuelei Wu, Jiakun Wang

**Affiliations:** 1 Institute of Dairy Science, College of Animal Sciences, Zhejiang University, Hangzhou 310058, China; 2 MoE Key Laboratory of Molecular Animal Nutrition, Zhejiang University, Hangzhou 310058, China; 3 College of Biological & Environmental Sciences, Zhejiang Wanli University, Ningbo 315100, China

**Keywords:** Agricultural Residues, Fungus, Solid-state Fermentation, *In vitro* Digestibility

## Abstract

**Objective:**

The experiment was conducted to evaluate the effects of four fungal pretreatments on the nutritional value of *Camellia* seed residues, and to evaluate the feeding value of pre-treated *Camellia* seed residues for ruminants.

**Methods:**

*Camellia* seed residues were firstly fermented by four lignin degrading fungi, namely, *Phanerochaete chrysosporium* (*P. chrysosporium*)-30942, *Trichoderma koningiopsis* (*T. koningiopsis*)-2660, *Trichoderma aspellum (T. aspellum*)-2527, or *T. aspellum*-2627, under solid-state fermentation (SSF) conditions at six different incubation times. The nutritional value of each fermented *Camellia* seed residues was then analyzed. The fermentation profiles, organic matter degradability and metabolizable energy of each pre-treated *Camellia* seed residue were further evaluated using an *in vitro* rumen fermentation system.

**Results:**

After 5 days of fermentation, *P. chrysosporium*-30942 had higher degradation of lignin (20.51%), consumed less hemicellulose (4.02%), and the SSF efficiency reached 83.43%. *T. koningiopsis*-2660 degraded more lignin (21.54%) and consumed less cellulose (20.94%) and hemicellulose (2.51%), the SSF efficiency reached 127.93%. The maximum SSF efficiency was 58.18% for *T. aspellum*-2527 and 47.61% for *T. aspellum*-2627, appeared at 30 and 15 days respectively. All the fungal pretreatments significantly improved the crude protein content (p<0.05). The *Camellia* seed residues pretreated for 5 days were found to possess significantly increased organic matter degradability, volatile fatty acid production and metabolizable energy (p<0.05) after the treatment of either *P. chrysosporium*-30942, *T. koningiopsis*-2660 or *T. aspellum*-2527. The fungal pretreatments did not significantly change the rumen fermentation pattern of *Camellia* seed residues, with an unchanged ratio of acetate to propionate.

**Conclusion:**

The fungi showed excellent potential for the solid-state bioconversion of *Camellia* seed residues into digestible ruminant energy feed, and their shorter lignin degradation characteristics could reduce loss of the other available carbohydrates during SSF.

## INTRODUCTION

The use of certain agricultural crop residues to provide energy and nutrients to animals is recognized as a sustainable way to support present levels of animal productivity [1]. However, the high lignin content and lower digestibility of agricultural crop residues limits their use as animal feed. The seed residues of tea-oil *camellia* (with the hulls) are one of the most common sources of lignification waste with several bioactive components such as protein, polysaccharides and polyphenols remaining [2].

The tea-oil *camellia* (*Camellia oleifera* [*C. oleifera*]) is one of the world’s four major woody oil trees, along with oil palm, olive and coconut. *Camellia* is a significant and widely promoted woody oil plant in China due to its high percentage of unsaturated fatty acids, and the oil of *C. oleifera* (tea-oil) has been called “Oriental Olive Oil” due to the similar oil composition between *C. oleifera* and olive [2]. Furthermore, *C. oleifera* is popular in the cosmetic and pharmaceutical industries because of the high level of saponin in the seed [3]. Indeed, more than 50% of cooking oil used in southern China, especially in Hunan Province, is tea-oil [4]. With *C. oleifera* playing a vital role in providing nutritional applications, biofuel productions and chemical feedstocks, up to 2 million tons of *C. oleifera* seeds are produced every year in China, and this number increases annually. After pressing for oil and extracting the tea saponin, the residues account for more than 60% of the total seed weight [5].

Traditionally, these residues are thrown away or burned up in the tea oil processing industry, which not only leads to environmental problems but also is a waste of bioresources. In consideration of the abundant bioactive substances in the oil cake and seed shell, many attempts have been made to exploit these byproducts. However, the tea oil fruit hull, which accounts for nearly 75% of the total byproducts, is a natural lignocellulosic biomass that consists of 26% to 27% lignin except for the bioactive substances [6]. Biological methods to remove lignin from plant residues are widely acceptable as they are environmentally benign. White-rot fungi, the most promising of the lignin degraders, have been studied for bioconversion of plant residues into nutritionally digestible animal feed [7,8]. Trichoderma (soft-rot fungi) have intricate complexities in both composition and function of the cellulolytic machinery but have been the best characterized lignin degraders [9]. White-rot and soft-rot fungi are the most prevalent culturable fungi. Solid-state fermentations (SSF) been proved to be the ideal technology for the practical, economical and environmentally-friendly bioconversion of agricultural wastes [10]. Therefore, the present study aimed to explore the effects of fungal SSF of *Camellia* seed residues on their nutrient profile and on *in vitro* gas and fermentation kinetics, with a view towards using them as a cleaner and more sustainable biological product for ruminant feed.

## MATERIALS AND METHODS

### Experimental design

The effects of four fungal pretreatments on the nutritional value of *Camellia* seed residues at six incubation times were measured with single factor analysis. The feeding value of pre-treated *Camellia* seed residues was then evaluated in an *in vitro* rumen fermentation system with the mixture of rumen fluid collected from three fistulated Hu sheep.

### Microorganism

One white-rot fungus, *Phanerochaete chrysosporium* (ACCC- 0942; *P. chrysosporium*-30942), was purchased from the Agri-cultural Culture Collection of China (ACCC). Three soft-rot fungi (*Trichoderma koningiopsis*-2660 [*T. koningiopsis*-2660], *Trichoderma aspellum*-2527 [*T. aspellum*-2527], and *Trichoderma aspellum*-2627 [*T. aspellum*-2627]) were donated by Dr. Chulong Zhang from College of Agriculture & Biotechnology, Zhejiang University. These fungi were grown and maintained on potato dextrose agar (PDA) containing the following (g/L): peeled potatoes 200; glucose 20; MgSO_4_·7H_2_O 1; KH_2_PO_4_ 1.5 with natural pH and at 4°C.

### Inoculum development

Erlenmeyer flasks (250 mL) containing 50 mL of PDA were used. Each flask was inoculated with four mycelial discs (each with a diameter of 8 mm) taken from the growing edges of 3-day-old fungal cultures. The flasks were incubated in an incubator shaker (HZ-9211K, Hualida, Taicang, China) at 150 rpm and 25°C for 36 h. The fungal suspension was used as seed inoculum for further large scale SSF of *Camellia* seed residues.

### Solid-state fermentation of *Camellia* seed residues

Each culture dish (150×30 mm) containing 10 g (dry weight) of *Camellia* seed residues (0.425 mm size) moistened with 10 mL of micronutrient solution (g/L: NaCl 0.5; MgSO_4_ 0.5; (NH_4_)_2_SO_4_ 3.0; KH_2_PO_4_ 1.0; CaCl_2_ 0.01; FeSO_4_·7H_2_O 0.01; MnSO_4_·7H_2_O 0.015; ZnSO_4_·7H_2_O 0.015; CoCl 0.01) was exposed to ultraviolet light for 30 min. The dishes inoculated with 2 mL of fungal suspension were used as treatments, and dishes without fungal suspension were used as controls simultaneously. All the dishes were incubated at 25°C in a biochemical incubator after being sealed with sealing films and incubated for 5, 10, 15, 20, 25, and 30 days. Triplicate dishes were used in parallel for each incubation period. After various fermentation periods were completed, the dishes were harvested, and oven dried at 65°C for 48 h and processed further for estimation of cell wall composition, crude protein (CP) content and *in vitro* digestibility.

### Chemical analysis

Samples of fermented *Camellia* seed residues were dried and ground through a 0.425-mm screen (Wiley mill, Arthur H. Co., Philadelphia, PA, USA) and analyzed for dry matter (DM) (#934.01), ash (#942.05), CP (#954.01), and ether extract (EE; #920.39) according to AOAC [11]. The neutral detergent fiber (NDF) [12], acid detergent fiber (ADF) and acid detergent lignin were analyzed using an ANKOM200 Fiber Analyzer Unit (ANKOM Technology Corporation, Macedon, NY, USA) [11]. The NDF was assayed with heat-stable alpha-amylase and with sodium sulfite in the neutral detergent solution. Both NDF and ADF were expressed without residual CP. The hemicellulose was calculated using the difference between NDF and ADF, and the cellulose was calculated using the difference between ADF and lignin.

### *In vitro* fermentation with the reading pressure technique

Samples (0.5 g) of each fermented *Camellia* seed residues were weighed into 120-mL serum bottles and deoxidized overnight inside of an anaerobic chamber with an atmosphere of 95% CO_2_ and 5% H_2_ (Vinyl Anaerobic Chambers, Coy Laboratory Products, Grass Lake, MI, USA). The next morning, before feeding, rumen fluid was collected from three fistulated Hu sheep (30.5±2.0 kg body weight), which were fed a total mixed ration (roughage:concentrate = 60:40) twice daily and strained through four layers of cheesecloth under continuous flushing with CO_2_. The maintance of rumen fistulated sheep, and procedure of rumen fluid collection were approved by the Animal Care Committee of Zhejiang University (Hangzhou, China). The three rumen fluid samples were combined in equal ratio and mixed as the inoculum. A mixture of a buffered medium [13] and the inoculum in a 9:1 ratio was prepared inside of the anaerobic chamber. Each 50 mL of this medium-inoculum mixture was dispensed to the serum bottles. Triplicate bottles were included for each of the fermented *Camellia* seed residues, and three blanks (rumen fluid only) were included simultaneously to correct the gas production (GP) values for gas release from endogenous substrates. All the bottles were incubated at 39°C for 48 h. The gas pressure in each culture bottle was recorded at 2, 4, 6, 9, 12, 24, 36, and 48 h using a pressure sensor (Ruyi, Shanghai, China). At the end of 48 h incubation, the fermentation process was stopped by swirling the bottles in ice. The bottles were then uncapped, the pH was measured using a pH meter (PB-10, Gottingen, Germany), and the contents of each bottle were collected for analysis of volatile fatty acid (VFA) concentrations using gas chromatography (GC-2010; Shimadzu Corp., Kyoto, Japan) and ammonia nitrogen using colorimetry [14,15].

### Calculations and statistical analysis

To describe the GP dynamics over time, the following equation was used to fit the data to the model described by Ørskov and McDonald [16]: GP = b*(1–e^−c*(t–λ)^), where GP = the accumulative GP (mL) at time t (h), b = the GP from the insoluble fraction (mL), c = the GP rate constant (%/h), and λ = the lag time.

The SSF efficiency was calculated following the formula of Moyson and Verachtert [17]: SSF efficiency = (loss of lignin/loss of hemicelluloses+cellulose). The organic matter digestibility (OMD) and metabolizable energy (ME) was estimated from the *in vitro* GP and CP content of the fermented *Camellia* seed residues with or without fungi. The OMD was calculated according to Menke and Steingass [18]. After the GP from the reading pressure techniques system transferred to the GP for the syringe system [19]. The OMD was calculated with the following formula: GP_24h_ for the syringe system (mL) = 1.2628 ×GP_24h_ for the RTP system (mL)+6.2592 [19]. OMD (% DM) = 24.59+0.7984×GP_24h_ for the syringe system (mL/200 mg DM) +0.0496×CP (g/kg DM) [18]. ME (MJ/kg DM) = −1.15+0.1600 ×OMD (% DM) [18]. The effects of fungal fermentation on the chemical composition of *Camellia* seed residues, SFF efficiency and *in vitro* GP, fermentation parameters, OMD and ME were analyzed using one-way analysis of variance, with multiple comparisons of means assessed by Tukey’s multiple range test at a significance level of 0.05. These analyses were conducted using R 3.2 (R Core Team, Vienna, Austria).

## RESULTS

### Compositional changes of *Camellia* seed residues

*P. chrysosporium*-30942, *T. koningiopsis*-2660, *T. aspellum*-2527, and *T. aspellum*-2627 grew well on *Camellia* seed residues under SSF conditions. The unfermented (control) *Camellia* seed residues were found to have the following: (g/kg DM) NDF, 791.2; ADF, 495.6; hemicellulose, 295.6; lignin, 279.2; cellulose, 216.4; CP, 121.1; and EE, 86.0.

**Figure 1:**
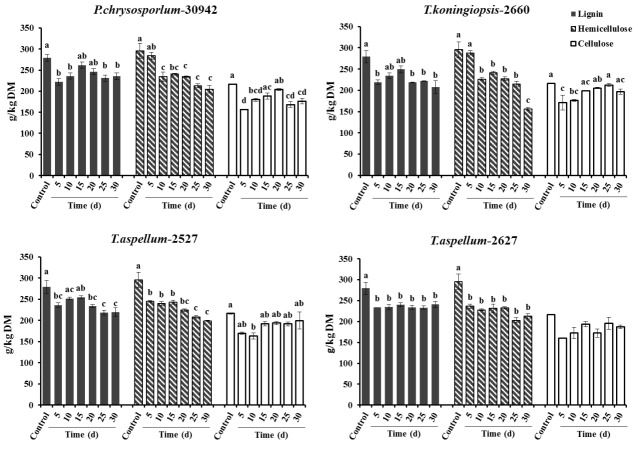
Lignin, hemicellulose and cellulose contents (g/kg DM) of control and fungal treated (*Phanerochaete chrysosporium*-30942, *Trichoderma koningiopsis*-2660, *Trichoderma aspellum*-2527, or *Trichoderma aspellum*-2627) Camellia seed residues under solid-state fermentation conditions. Bars indicate the standard error. ^a–d^ Different letters represent significant differences (p<0.05). DM, dry matter.

**Figure 2:**
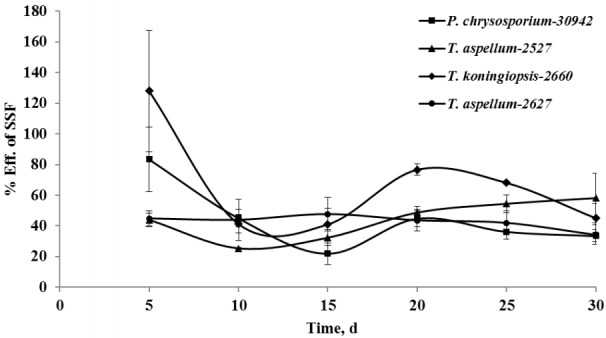
Lignin loss compared to hemicellulose and cellulose losses during the solid-state fermentation (SSF) process with the treatments of *Phanerochaete chrysosporium*-30942, *Trichoderma koningiopsis*-2660, *Trichoderma aspellum*-2527, or *Trichoderma aspellum*-2627. Efficiency of SSF is the derived ratio from the values of these components; Efficiency of SSF = (loss of lignin/loss of hemicellulose+cellulose)×100. Bars indicate standard error.

After 5 days of fermentation during the 30 days of incubation, *P. chrysosporium*-30942 could have had higher degradation in lignin (20.51%) but consumed less hemicellulose (4.02%) and was accompanied by a 27.54% decrease of cellulose content (Figure 1). The lignin and cellulose content of 5-day fermented *Camellia* seed cakes were significantly lower than controls (p< 0.05), while the hemicellulose content was significantly different until 10 days of incubation (Figure 1, p<0.05). The cell wall degradation profile of *Camellia* seed residues showed that the P*. chrysosporium*-30942 degraded lignin at a faster rate compared to cellulose until the 20th day of fermentation. The percent of SSF efficiency, which is an index to measure the amount of lignin degradation at the expense of carbohydrate content loss, was found to reach the maximum level (83.43%) at the 5th day of fermentation (Figure 2).

A similar degradation pattern was observed for the *T. koningiopsis*-2660 (Figure 1). However, *T. koningiopsis*-2660 degraded more lignin (21.54%) and consumed less cellulose (20.94%) and hemicellulose (2.51%) at the same time after the 5th day of fermentation, and the percent of SSF efficiency reached 127.93% (Figure 2). These results indicate that *T. koningiopsis*-2660 more efficiently degraded lignin compared to hemicellulose and cellulose consumption in a shorter time.

The other two fungi tested, *T. aspellum*-2527 and *T. aspellum*-2627, showed a faster rate of lignin and hemicelluloses degradation compared to the degradation of cellulose (Figure 1). Both significantly reduced the lignin and hemicellulose content in the 5 days fermented *Camellia* seed residues (p<0.05), and the content of cellulose was reduced significantly until 10 days of fermentation with the treatment of *T. aspellum*-2627 (p<0.05). Simultaneously, there was no significantly change in cellulose content of the *T. aspellum*-2527 fermented *Camellia* seed residues (Figure 1). The maximum percent of SSF efficiency was 58.18% for *T. aspellum*-2527 and 47.61% for *T. aspellum*-2627, appeared at 30 and 15 days respectively (Figure 2).

**Figure 3:**
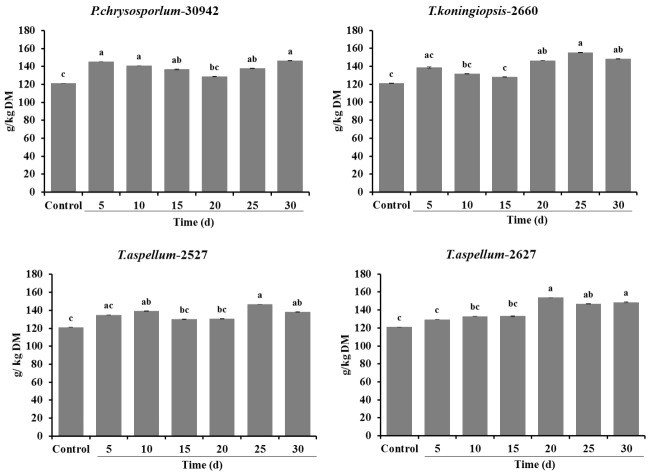
Crude protein content (g/kg DM) of control and fungal-treated (*Phanerochaete chrysosporium*-30942, *Trichoderma koningiopsis*-2660, *Trichoderma aspellum*-2527, or *Trichoderma aspellum*-2627) *Camellia* seed residues under solid-state fermentation conditions. Bars indicate the standard error. ^a–c^ Different letters represent significant differences (p<0.05). DM, dry matter.

The CP content of all *Camellia* seed residues increased significantly (p<0.05) with the treatment of *P. chrysosporium*-30942 (5, 10, 15, 25, 30 days of incubation), *T. koningiopsis*-2660 (20, 25, 30 days of incubation), *T. aspellum*-2527 (10, 25, 30 days of incubation) and *T. aspellum*-2627 (20, 25, 30 days of incubation) during 30 days of SSF (Figure 3).

### *In vitro* evaluation of fermented *Camellia* seed residues

Considering the percent of SSF efficiency, CP and degradation pattern at the same time, *Camellia* seed residues fermented for 5 days were found to have high availability of carbohydrate-based nutrients as well as energy content. An *in vitro* rumen fermentation test was used to evaluate the changes of fermentation parameters of *Camellia* seed residues after the pretreatment of fungi for 5 days.

**Figure 4:**
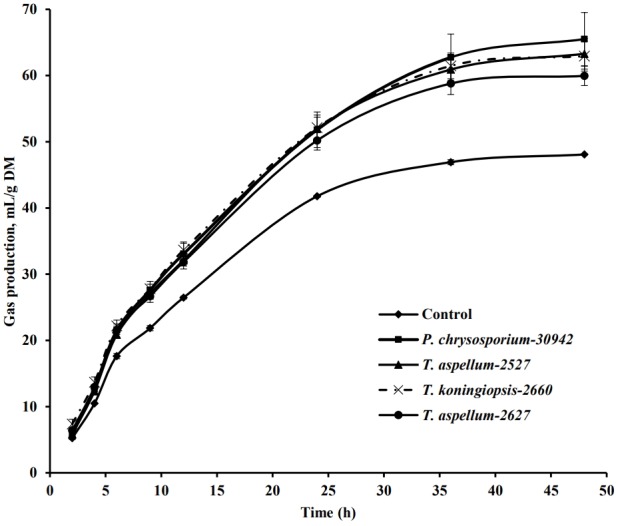
*In vitro* gas production pattern during 48 h of incubation of *Camellia* seed residues that have been pretreated by fungus for 5 days. Bars indicate the standard error. DM, dry matter.

The *in vitro* rumen GP (m/g DM substrate) of *Camellia* seed residues was affected by the fungal pretreatment during 48 h of incubation (Figure 4). The GP from fermented *Camellia* seed residues increased significantly (p<0.05) compared to the control at 6, 9, 12, 24, and 48 h of incubation. With the analysis of dynamic fermentation parameters, the GP from the insoluble fraction of *Camellia* seed residues (b value in Table 1) was increased significantly (p<0.05) by the *P. chrysosporium*-30942, *T. koningiopsis*-2660, and *T. aspellum*-2527 treatments, with increases of 39.72%, 33.03%, and 34.00%, respectively. While the rate of GP (c value) was not increased significantly (p = 0.15) by the four fungal treatments.

Further analysis showed that the pH value after 48 h of *in vitro* rumen fermentation for fermented *Camellia* seed residues was significantly reduced when pretreated with *P. chrysosporium*-30942, *T. koningiopsis*-2660 and *T. aspellum*-2527 (p<0.01, Table 1), but the pH remained higher than 7.0. All the treatments did not significantly affect the concentration of ammonia nitrogen. Fermented *Camellia* seed residues pretreated by either *P. chrysosporium*-30942, *T. koningiopsis*-2660, *T. aspellum*-2527 or *T. aspellum*-2627 produced significantly higher amounts of total VFA (TVFA, p<0.01), acetate (p<0.01) and butyrate (p<0.01) than the controls (Table 1). Propionate production from fermented *Camellia* seed residues pretreated by *T. koningiopsis*-2660 was also significantly higher than controls (p< 0.05, Table 1). All the treatments did not significantly affect the ratio of acetate to propionate (Table 1).

**Figure 5:**
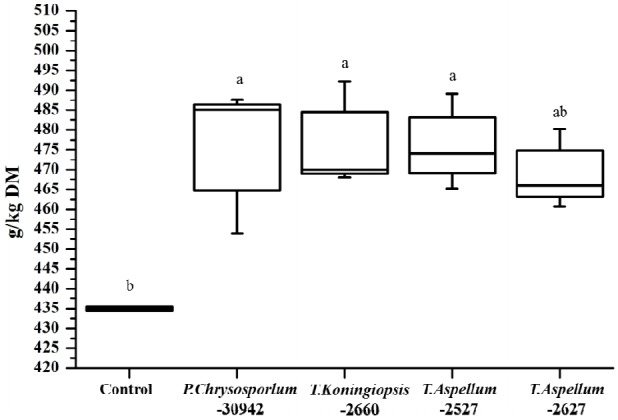
Digestibility of organic matter after 24 h of *in vitro* rumen fermentation of fungal fermented *Camellia* seed residues. ^a,b^ Different letters represent significant differences (p<0.05). DM, dry matter.

**Figure 6:**
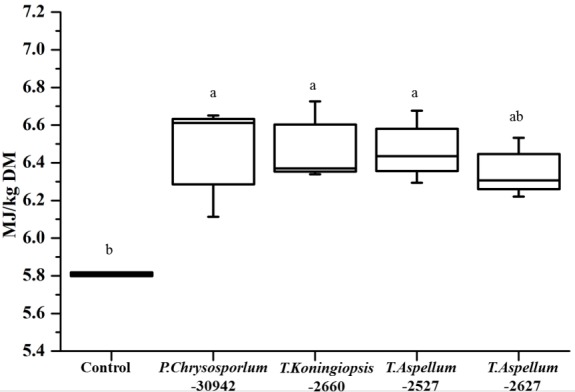
Metabolizable energy of fungal fermented *Camellia* seed residues after 24 h of *in vitro* rumen fermentation. ^a,b^ Different letters represent significant differences (p<0.05). DM, dry matter.

The OMD of *Camellia* seed residues was 434.9 g/kg DM and was increased significantly by *P. chrysosporium*-30942, *T. koningiopsis*-2660, and *T. aspellum*-2527, with increases of 9.34%, 9.62%, and 9.12%, respectively (Figure 5). The ME of *Camellia* seed residues was 5.81 MJ/kg DM, and this was significantly improved by the fungal treatments of *P. chrysosporium*-30942, *T. koningiopsis*-2660, and *T. aspellum*-2527 (Figure 6). The ME was increased to 6.46, 6.48, and 6.47 MJ/kg DM, respectively.

## DISCUSSION

High lignin content in agricultural crop residues generally hinders the maximum accessibility of carbohydrates to the gut microorganisms of ruminants, which discourages their direct use as animal feed because of poor digestibility and utilization [6]. Pre-treating the agricultural crop residues with fungi as a biological alternative has recently been the focus of much research because the fungal lignocellulolytic enzymes could break down the polysaccharide-lignin complex and improve the accessibility of digestible biomass. Further, the fungi could also serve as a good source of protein for animal growth. The degradation of structural carbohydrates and lignin is an important factor that would affect the digestibility improvement of agricultural crop residues after SSF [20]. Various fungi, such as *Bjerkanderaadusta*, *Ceriporiopsis subvermispora*, *Ganoderma* sp., *Lentinula edodes*, *Pleurotus* sp., *Phlebia brevispora*, and *Tinea versicolor* [*T. versicolor*], are known for their degradation of lignin and have been proven to bio-convert plant residues into nutritionally digestible animal feed in SSF conditions. However, most of these fungi degrade lignin at a relatively slow rate and are accompanied by the degradation of cellulose and hemicelluloses [7,8,21]. *Ganoderma* sp. needs 49 days to degrade 11% of lignin, while *Pleurotus* sp. and *T. versicolor* take 30 days to degrade 37% and 31% of lignin, respectively [7,8,21]. When wheat straw was fermented by *P. ciliatus*, along with lignin, the greatest loss in contents was for cellulose and hemicelluloses [22]. White rot fungus, *P. chrysosporium*, could degrade cellulose and hemicellulose much faster than lignin in paddy straw, but an almost equal amount of the three fibers were degraded when incubated in wheat straw, while more lignin was degraded than other fibers during the SSF of sugarcane bagasses [23,24]. In our study, 83.43% and 127.93% of SSF efficiency were observed in the *P. chrysosporium*-30942 and *T. koningiopsis*-2660 pretreatments, respectively, by the 5th day of SSF, suggesting an efficient degradation of lignin in *Camellia* seed residues in a shorter period. This efficiency prevented the loss of cellulose and hemicellulose, which act as important energy sources for ruminants.

Consistent with previous studies [21,25], the pretreatment of *Camellia* seed residues with *P. chrysosporium*-30942, *T. koningiopsis*-2660, *T. aspellum*-2527, or *T. aspellum*-2627 increased the *in vitro* digestibility and available energy (VFA) after 5 days of fermentation. The fermentation parameters were positively related to the SSF efficiency. Fat-rich feeds should be used with caution in ruminant animals for the negative effect on fiberlytic bacteria. Though the EE of the pretreated *Camellia* seed residues was not measured in the current study, and it increased in *Trichoderma viride* treated peanut hulls [26], the increased VFA, and unaffected molar proportion of acetate and propionate of fungal treatments in the current study, suggesting that there were no negative effects on fiberlytic bacteria and no change in the availability of hydrogen gas for the synthesis of methane. These fermentation parameters suggest that fungal SSF might have enhanced the utilization of *Camellia* seed residues in the rumen.

The pH is an important parameter reflecting the ruminal environment. In the current study, the decreased pH during rumen fermentation of pre-treated *Camellia* seed residues may have been caused by the production of acidic compounds during the fungal SSF of complex lignins and sugars. The lower pH during fungal degradation of lignocellulosics has been reported in previous studies, because a mixture of acids would be produced during the second oxidation step of lignin degradation [23]. However, the values in the present study were all within the normal range (>6.3) for optimum rumen metabolism [27].

The improvement in CP content following fungal degradation of agricultural crop residues has been reported by many previous studies [7,8]. Ammonia N serves as an indicator of protein degradation in the rumen, where it is also the major source of microbial protein synthesis [28]. Wanapat and Pimpa [29] reported that the optimum level of ammonia N in rumen is 0.5 to 0.8 mg/L in the mixed rumen culture. The improved capture of nutrients during SSF would cause more of the carbon source to be directed to produce microbial protein [28]. As the gas was produced by microbiota utilizing carbohydrate, and there was enough nitrogen in the *in vitro* buffer; the *in vitro* rumen fermentation culture is not a good model to evaluate the effects of protein on rumen fermentation. Though the effects of increased protein were calculated into the OMD and ME, it is better to estimate the contributions of fungal protein to meat or milk *in vivo*.

The CP and ME of fungal fermented *Camellia* seed residues were approximately 140 g/kg DM and 6.45 MJ/kg DM. The CP and ME of the cold season gramineous forage grass were 120.3 g/kg DM and 7.78 MJ/kg DM, respectively [30]. The CP and ME of wheat straw was 51.8 g/kg DM and 6.02 MJ/kg DM, respectively [30]. Clearly, the fungal fermented *Camellia* seed residues were more effective in terms of CP, and the ME was also reasonable.

The fungal fermented *Camellia* seed residues were acquired with SSF using culture dish. Though the use of tray fermenters in large-scale production is limited as they require a large operational area, no forced aeration or mixing of the residues during the fermentation tended to lower labour intension. Furthermore, 5 days’ fermentation of *Camellia* seed residues at 25°C without additional carbohydrate or CP is easy and economical to carry out in farm at the harvest season of *Camellia* seed.

## CONCLUSION

In summary, the present study showed that fungal treatment can improve the nutritional quality of *Camellia* seed residues. Because of their fast growing and selective lignin degrading characteristics, *P. chrysosporium*-30942, *T. koningiopsis*-2660, and *T. aspellum*-2527 can be potential candidates for the solid-state bioconversion of *Camellia* seed residues into digestible ruminant energy feed. Five days is a suitable fermentation time for that characterized by higher OMD, VFA production and ME. The optimal fermentation conditions and feeding efficiency should be examined in the future for commercial production and applications.

## Figures and Tables

**Table 1:** *In vitro* rumen fermentation profile of fungal fermented *Camellia* seed residues

Items	Control	*P. chrysosporium-30942*	*T. koningiopsis-2660*	*T. aspellum-2527*	*T. aspellum-2627*	SEM	p-value
GP_48h_ ( mL/g DM)	48.08^b^	65.50^a^	62.93^a^	63.29^a^	59.95^ab^	2.53	0.02
b (mL)	50.86^b^	71.06^a^	67.66^a^	68.15^a^	64.27^ab^	2.98	0.02
c (mL/h)	3.47	4.02	4.12	4.06	4.03	0.15	0.15
λ (h)	0.43	0.34	0.10	0.47	0.26	0.14	0.40
pH	7.25^a^	7.04^c^	7.04^c^	7.11^bc^	7.18^ab^	0.02	<0.01
Ammonia nitrogen (mg/L)	2.52	2.45	2.54	2.55	2.50	0.04	0.35
Volatile fatty acids (mmol/L)	32.18^b^	36.78^a^	37.95^a^	36.02^a^	35.94^a^	0.52	<0.01
Acetate (mmol/L)	21.92^b^	25.04^a^	25.71^a^	24.78^a^	24.38^a^	0.34	<0.01
Propionate (mmol/L)	7.07^b^	7.90^ab^	8.22^a^	7.54^ab^	7.73^ab^	0.19	0.02
Butyrate (mmol/L)	1.96^b^	2.57^a^	2.71^a^	2.44^a^	2.56^a^	0.07	<0.01
Isobutyrate(mmol/L)	0.41^a^	0.39^ab^	0.40^ab^	0.38^b^	0.40^ab^	0.01	0.03
Valerate (mmol/L)	0.38	0.42	0.42	0.41	0.41	0.01	0.06
Isovalerate(mmol/L)	0.45	0.47	0.49	0.47	0.47	0.01	0.05
Acetate:propionate	3.10	3.17	3.13	3.29	3.16	0.07	0.38

SEM, standard error of the mean; GP, gas production; b, the GP from the insoluble fraction; c, the GP rate constant; λ, the lag time.

a–c Different superscripts in a row represent significant differences (p<0.05).
